# LIVING WITH YOUNG ONSET DEMENTIA IN RESIDENTIAL CARE: A SCOPING REVIEW

**DOI:** 10.1093/geroni/igae098.4014

**Published:** 2024-12-31

**Authors:** Linda Johansson, Therése Bielsten

**Affiliations:** School of Health and Welfare, Jönköping University, Jönköping, Jonkopings Lan, Sweden; Jönköping University, Jönköping, Jonkopings LanSweden

## Abstract

About 3.9 million people live with Young Onset Dementia (YOD) worldwide. As dementia progresses, there may be a need for transition to residential care. It can be challenging for persons with YOD to access age-appropriate services, even though they might have different needs compared to persons with late-onset dementia. This scoping review aimed to present the experiences of people with YOD living in residential care facilities. Peer-reviewed research articles focusing on either the perspective of people with dementia onset before the age of 65 living in residential care or their family members were included. A literature search in MEDLINE, CINAHL, PsycINFO, Scopus, ProQuest, and Web of Science conducted in December 2023 yielded 13 qualitative studies from Europe, North America, and Australasia. Four studies included experiences of persons with YOD, three included experiences of family members, and six had a mix of participant experiences. In total, 90 (Md = 9) persons with dementia and 292 (Md = 17) family members participated. A synthesis of findings indicates that a home-like environment and age-adjusted activities based on the interests of the individual were highly valued. This review provides a first step towards understanding what person-centered care means for people with YOD in residential care. However, even though people with YOD can communicate their experiences and needs, there were few studies focusing on their perspectives. To improve care, further research needs to focus on the voices of individuals with dementia.

